# Reduction in Ultrasonic Vocalizations in Pups Born to Rapid Eye Movement Sleep Restricted Mothers in Rat Model

**DOI:** 10.1371/journal.pone.0084948

**Published:** 2014-01-13

**Authors:** Kamalesh K. Gulia, Niraj Patel, Arathi Radhakrishnan, Velayudhan Mohan Kumar

**Affiliations:** Sleep Research Laboratory, Comprehensive Center for Sleep Disorders, Sree Chitra Tirunal Institute for Medical Sciences and Technology, Trivandrum, Kerala, India; Imperial College London, United Kingdom

## Abstract

The effects of rapid eye movement sleep restriction (REMSR) in rats during late pregnancy were studied on the ultrasonic vocalizations (USVs) made by the pups. USVs are distress calls inaudible to human ears. Rapid eye movement (REM) sleep was restricted in one group of pregnant rats for 22 hours, starting from gestational day 14 to 20, using standard single platform method. The USVs of male pups were recorded after a brief isolation from their mother for two minutes on alternate post-natal days, from day one till weaning. The USVs were recorded using microphones and were analysed qualitatively and quantitatively using SASPro software. Control pups produced maximum vocalization on post-natal days 9 to 11. In comparison, the pups born to REMSR mothers showed not only a reduction in vocalization but also a delay in peak call making days. The experimental group showed variations in the types and characteristics of call types, and alteration in temporal profile. The blunting of distress call making response in these pups indicates that maternal sleep plays a role in regulating the neural development involved in vocalizations and possibly in shaping the emotional behaviour in neonates. It is suggested that the reduced ultrasonic vocalizations can be utilized as a reliable early marker for affective state in rat pups. Such impaired vocalization responses could provide an important lead in understanding mother-child bonding for an optimal cognitive development during post-partum life. This is the first report showing a potential link between maternal REM sleep deprivation and the vocalization in neonates and infants.

## Introduction

Sleep restriction during pregnancy is an emerging concern that can have serious implications on the child's emotional development. Offspring born to sleep-compromised mothers exhibited expressions of anxiety disorders and an increased prevalence of cognitive deficits [1]–[4]. The rapid eye movement (REM) component of sleep is reduced during the last trimester of pregnancy even in healthy women [5]–[8] and in rats [9]–[10]. The effects of further reduction in REM sleep during pregnancy on the neural development of the newborn are largely unknown.

Vocalization is a potential means of communication between mother and child, and it is an important aspect in the developmental milestone of babies [11], [12]. After birth, cry is the first acoustic signal elicited by human neonates to express distress of being outside the mother's cosy womb. Neonates utilize ‘cry’ as a principal means to express their pervasive discomfort to isolation-induced stress [11]–[13]. Rodent pups emit audible calls primarily during tactile contact with their mother during nursing that assist the mother in establishing and maintaining care-giving responses [14]. In addition, these pups also produce distress calls in the form of ultrasonic vocalizations (USVs) when isolated from their mother and littermates.

Isolation in early post-partum weeks nearly imposes life threatening situations, as pups are born blind, deaf, and hairless with under-developed thermoregulatory capacity, and a limited capacity to move. USVs possibly signal stress/anxiety for an imperative retrieval by the dams [15]–[18]. The USVs in rat pups are recognized as homologous to cries of human infants [12], [19]–[22]. Although both types of vocalizations (USVs and audible cries) in pups have communicative value per se, the USVs probably reflect their anxiety traits/affective state to a larger extent [23]–[25]. In contrast to the USVs emitted by adults, which have distinct acoustic features [22], [26]–[28], the increased or reduced vocalizations in pups are expressions of anxiety [23]–[25], [29]. The sustained reduction in vocalization could even reflect despair and desolation during early development [30].

Recent reviews have indicated an overall decline in cognitive function, including alertness, attention and memory processing, after sleep restriction [31]–[34]. The magnitude of deficits in cognitive functions is dependent on several factors like magnitude of sleep loss, emotional state, stress at work etc, and these deficits accumulate over time due to adaptation to sensation of sleepiness [31]. These reports attempt to explain the effects of sleep deprivation on cognition in adults, but no study has looked into the association between sleep loss especially REM sleep restriction (REMSR) during pregnancy and its consequences on the newborn.

As we now know that the USVs in pups reflect emotional expression of despair and desolation during early development, these signals could be studied to assess the possible effects of REMSR on the newborns. Since the neurodevelopment is incomplete at birth, it is essential to record USVs till day 21 to gather a complete developmental profile on different postnatal days. Hence, the present study was undertaken to investigate the effects of maternal REMSR during the third trimester on the USV profiles, using isolation paradigm, in pups from postnatal day (pnd) 1 of birth to weaning, i.e. pnd 21.

## Materials and Methods

### Animals

The study was carried out on pups born to the mothers that were REM sleep restricted during the 3^rd^ trimester. Adult nulliparous female Wistar rats (body weight 220–240 g), after mating with male rats of similar age, were marked for pregnancy on the basis of vaginal plug formation. After confirmation of pregnancy, females were housed individually in polystyrene cages at controlled temperature (26±1°C) and light-dark schedule of 12 h (lights on at 06:00 h). Food and water were provided *ad libitum* throughout the experiment.

The pregnant rats (n = 10) were randomly divided into two groups. REM sleep was restricted in the first group of rats (for 22 h) from 11 AM to 9 AM on the next day, starting from gestational day 14 until day 20, using standard single platform method [35]. The platform provided was of 7 cm diameter in the REMSR group, whereas platform of larger diameter (16 cm) was used in sham control group. Water level was kept 3 cm below the surface of platform. After completion of the sleep restriction protocol, females were transferred to their home cages for parturition. Litter size (n = 6) was kept uniform by culling the remaining pups on day one of parturition in all the animals in both the groups.

### Ultrasonic vocalization measurement

The USVs of pups were recorded on brief isolation from their mothers. The control group pups provided data about the natural course of development of vocalizations on different postnatal days. These were compared with USVs of pups of REMSR group mothers. The USVs generated by individual pups on isolation were recorded on alternate days from pnd 1 – 21 for a period of 2 min at room temperature of 28±1°C. The ambient temperature within the recording site was monitored during 2 min of USV recording using FLUKE True-rms digital multimeter (Model 287/289; USA) with K type thermocouple containing chromal-alumel probe. The home cage with the dam and the pups was carried to the testing room. It took 8 to 10 seconds to isolate the pups, one at a time from the mother, to a glass beaker (with cotton bedding to reduce artefacts) kept inside a sound attenuated recording chamber, to test the effect of maternal REMSR on USVs. Prior testing was done to ensure that the USVs produced by the isolated pup do not reach the home cage, kept 2 meters away, where the other pups remained with the mother. After USV recordings, the pups were taken for retrieval test.

The USVs were recorded using a microphone (CM16/CMPA, Avisoft Bioacoustics, Berlin, Germany) placed 10 cm above the pups. The microphone was connected to a pre-amplifier (Avisoft UltrasoundGate 416H, Avisoft Bioacoustics) and digitized sonograms were stored in computer. Recorded USVs were analysed quantitatively and qualitatively using Avisoft SASLab Pro software (Version 5.1). The Fast Fourier Transform (FFT) was conducted to generate the spectrogram (FFT length 256 points, frame size of 100% in Flat top window with temporal resolution of 75%). The spectrogram was produced at frequency resolution of 977 Hz and a time resolution of 0.25 ms.

On each test day, the parameters analyzed included (1) calling rates i.e. number of calls/min, (2) call types, (3) duration of calls, (4) total time spent in calling, (5) carrier or fundamental frequency (F_0_) of calls, (6) amplitude for loudness and (7) temporal profile in call numbers (distribution of calls over first and second minute).

### Retrieval test

Retrieval test was done to assess the maternal response to USVs during first week. The retrieval test was conducted between 9–10 am in the home cage with fresh bedding. After the mother (without pups) was habituated in the cage for 5–7 minutes, all the six pups were placed scattered in cage, and were video monitored. The latency to retrieve the first pup and time taken for retrieval of all pups in five minute test period were noted.

### Body temperature

The body temperatures of pups were also observed in three different states a) when these were confined to the mother's crouch b) huddled in the nest with other pups and c) when they were lying scattered in the cage during the initial week. IR38 non-contact Thermolert thermometer was used for non-invasive measurement of the body temperature (CONSTANT Health Care, Australia). It had memory for ten data that ensured quick assessment of the temperature of all the six pups with minimal disturbance to them.

The study was approved and performed in accordance with the guidelines laid down by the Institutional Animal Ethics Committee of the Sree Chitra Tirunal Institute for Medical Sciences and Technology, Trivandrum, Kerala. Postpartum mother and pups were disturbed very minimally during the experiment.

### Statistical analysis

One way Analysis of Variance (ANOVA) with repeated measures, and post-hoc comparison with Bonferroni correction were conducted to compare the natural developmental profiles of the USV number (calling rate) and parameters (duration of call, F_0_) in the control and REMSR group over different postnatal days. Non-parametric analysis (Mann Whitney U test) was used to analyze differences in parameters between two groups. The level of significance was set at *p*<0.05 for all comparisons.

## Results

### Rate of Ultrasonic vocalization

**Figure 1:**
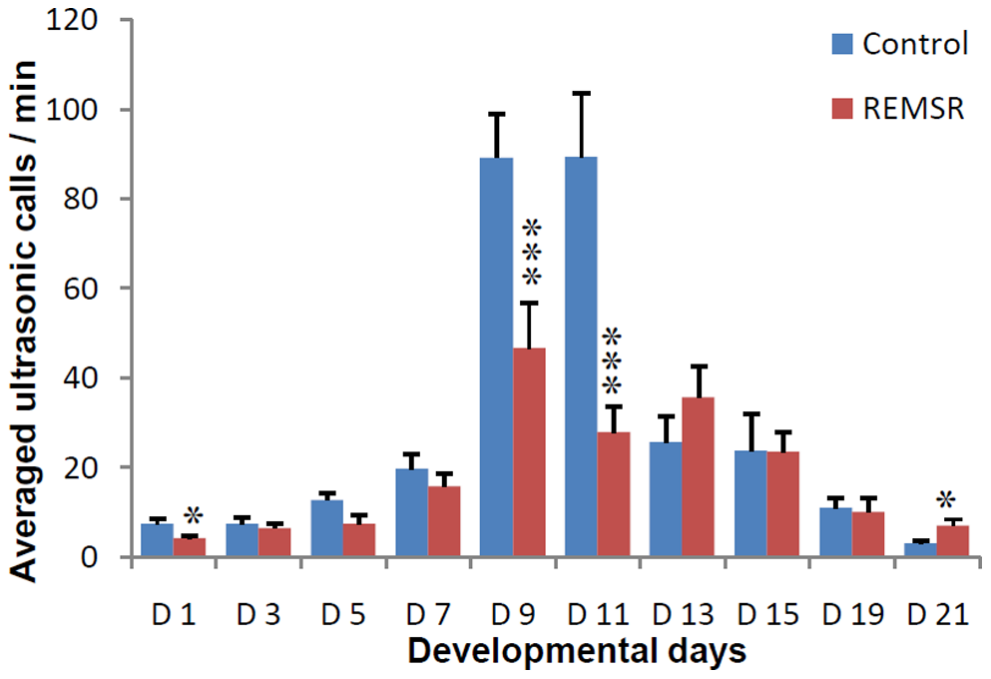
Average no of USVs/min in pups after maternal separation (N = 19 each). Vocalizations are presented as averaged calls/min ± SEM (Standard error of mean) in the Y axis and different developmental days on X axis. ‘D’ refers to days, Control values are represented in blue bars while the REMSR group values are shown in brown bars. The comparison of values between Control v/s REMSR group is depicted in ‘*’. The significance values are given as ‘*’ for p<0.05, ‘***’ for p<0.001.

A total of 17991 ultrasonic calls were obtained from pups on various days. The total numbers of USVs per min were averaged for each developmental day studied in 19 male pups, in each group, obtained from 10 litters. The intra and inter group multiple comparisons were made for all days for pups in control and REMSR group. The total numbers of calls in the pups of the control group remained low during initial days (Day 1–5). These were increased on pnd 7 and reached to highly significant values on peak days 9 and 11 (Figure 1). Thereafter, the total number of calls was reduced, reaching to a very low value on day 21.

The USVs of the pups born to REMSR mothers were half or lower (46.6±10.2 and 27.8±5.8) than the control values (89.2±9.7 and 89.2±14.2) on peak vocalization days of 9 and 11 respectively. A comparison of the total USVs in the pups born to REMSR mothers with the pups of the control group showed a reduction in calling rate on most of the initial days, though statistical significance was observed only on days 1, 9 and 11 (Figure 1). Thereafter, the number of calls of pups born to REMSR mothers were not only comparable; it was even higher than pups of the control group on day 21.

### Types of ultrasonic vocalizations

**Figure 2:**
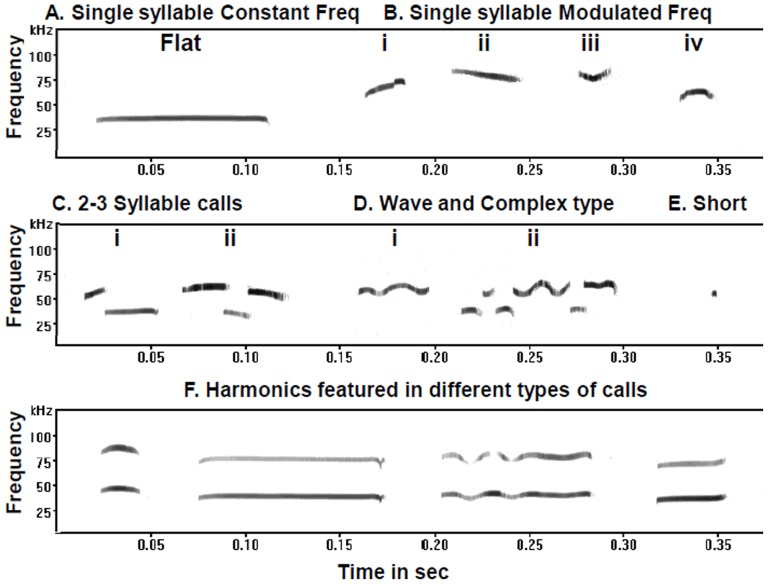
Sonograph of different call types produced by pups in the isolation paradigm. The frequency is shown in kHz on the Y axis and time scale is expressed in sec on the X axis. The CF and MF refers to constant and modulated frequency respectively. In single syllable MF category, numeral i, ii, iii and iv denotes Upward, Downward, U-shaped and Inverted U-shaped calls.

Individual calls were labelled on the basis of shape and sonographic features, primarily in accordance with the prevailing classification [17], [36]. USVs were grouped into five categories on the basis of number of syllables, frequency modulation and duration (Figure 2). The calls having constant frequency (CF) with a variation of ±5 kHz from start to end were designated as Flat calls (Figure 2A). The single syllable calls having simple frequency modulation were grouped into Single syllable frequency modulated (MF) category (Figure 2B) which consisted of upward sweep (Figure 2A-i), downward sweep (Figure 2A-ii), U-shaped (Figure 2A-iii), and inverted U shaped (Figure 2A-iv) calls. In 3^rd^ category (Figure 2C), all two or three syllable calls were clustered. All the calls displaying complex frequency modulation were grouped together as Wave and Complex type (Figure 2D). Calls with durations of less than 5 ms were taken as short calls (Figure 2E). The calls in the form of harmonics or additional overtones over the fundamental call were also common, which were referred to as harmonics (Figure 2F). The percentage of calls in the form of harmonics was calculated separately from the total calls/day, irrespective of the types.

### Call types on different days in control and REMSR group pups

**Figure 3:**
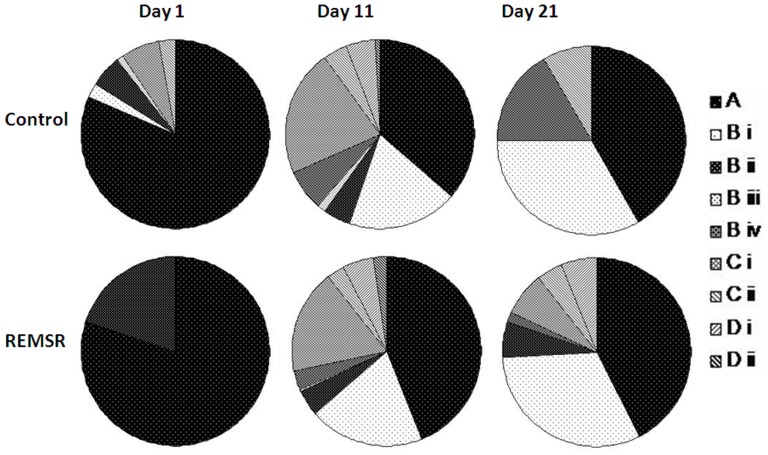
Distribution pattern of isolation calls during different developmental days in the control and REMSR group. The percent distribution of various calls grouped in four categories obtained on isolation at postnatal day 1, 11 and 21 is shown in pie diagram for pups in the control and REMSR group. A denotes category consisting of flat calls; B having upwards (i), downwards (ii), U shaped (iii), and inverted-U (iv); C is sum of 2 and 3 syllable and D contains wave and complex calls.

The distribution of call types on postnatal days 1, 11 and 21 are shown in Figure 3. On pnd 1, call types were minimal (flat and downwards) in the pups of REMSR mothers. From pnd 9 (the peak calling day) onwards all the call types were found in both the groups; however, there were significant variations among them.

**Figure 4:**
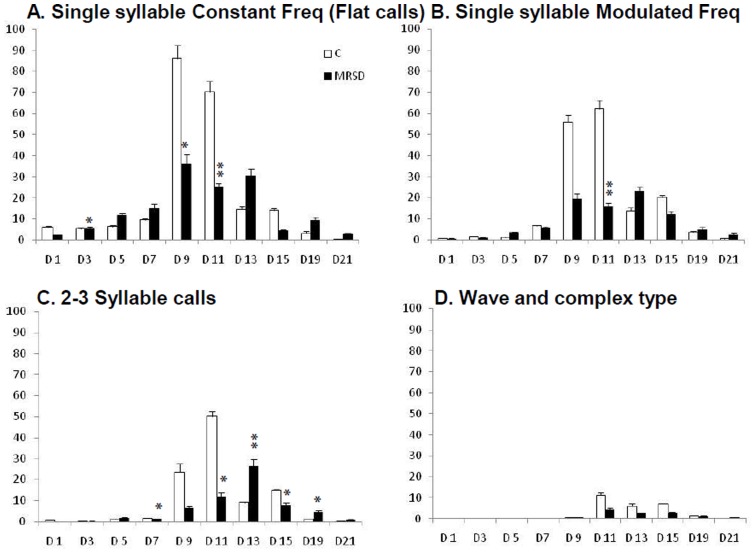
Distribution pattern of isolation calls grouped in four categories in the control and REMSR group. Distribution pattern of isolation calls grouped in four categories during different developmental days in the control and REMSR group. Developmental days are shown in horizontal axis and averaged numbers of calls are shown in Y axis.

In the control group pups during the initial postnatal week, the predominant flat calls were frequent, ranging from 80% on pnd 1 to 60% on pnd 7. They decreased during the later developmental days when the other call types in higher frequency modulation and complexity increased in number (Figure 4). A comparison of the call types in different categories between the control and REMSR groups was performed. Of all the categories of calls, flat calls consisting of constant frequency were more frequent in comparison to remaining call types in both the groups (Figure 4). Pups in REMSR group made significantly lower number of flat calls on days 3, 9 and 11 in comparison to the control group, even though calls in this group were also lower (not statistically significant) on days 1 and 15 (Figure 4A). On pnd 5, the vocalization in the flat, MF single syllable and 2–3 syllable call types were higher in number.

There was a significant decrease in single syllable MF calls on pnd 11 in REMSR group (Figure 4B). However, a comparison of calls within this group showed that the downward calls were higher on day 5 in the pups of REMSR mothers, whereas upward calls were higher on pnd 9 and 11. Inverted-U calls remained higher on pnd 11 in control group pups (not displayed in Figure 4).

**Figure 5:**
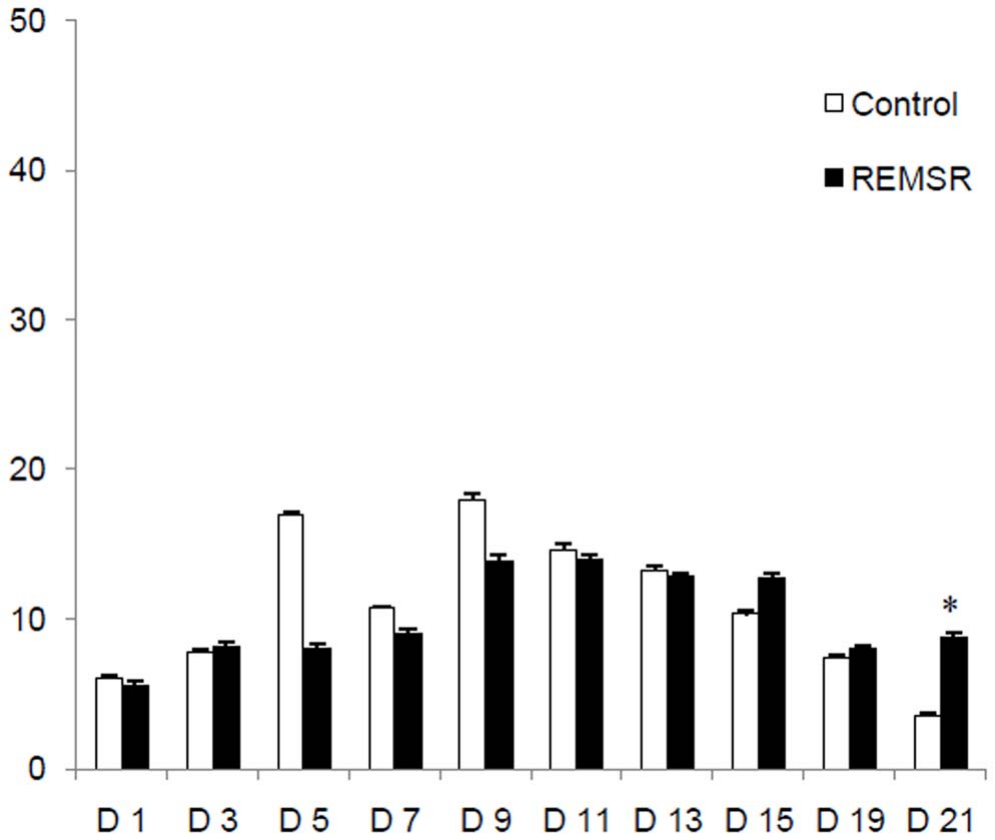
Distribution pattern of dot calls during different developmental days in the control and REMSR group. Distribution pattern of dot calls during different developmental days in the control and REMSR group. Developmental days are shown in horizontal axis and averaged numbers of calls are shown in Y axis.

A shift in the peak calling days of 2-3 syllable USVs of REMSR group towards the end of the second week resulted in significant differences between the two groups on post-natal days 7, 11, 13, 15 and 19 (Figure 4C). The 2 syllable calls remained higher on pnd 7, and 3 syllable calls were higher on pnd 13 in the pups of the REMSR mothers. Wave and complex calls could be observed only after pnd 7 and their numbers were low in both the groups of pups during the observed days (Figure 4D). No significant variation was observed in dot calls between the control and the REMSR groups, except on pnd 21 (Figure 5).

### Call durations (Total and averaged)

Variations in the mean duration of different call types between the control and the REMSR groups are tabulated for various developmental days (Table 1). The highest durations were observed on peak days, i.e. pnd 9 or 11, in the control group pups. On pnd 5, flat calls (category A) in the REMSR group were significantly longer in comparison to the control group ones. Downwards (B ii) and 2–3 syllable calls (C) were shorter in the REMSR group on days 9 and 11; however, statistical significance was reached only on day 11. The longer duration calls in upward, downward, U-shaped and 2–3 syllables were also made by the pups of the REMSR mothers on pnd 5.

**Table 1:** Mean durations of individual call types in different categories between control and REMSR groups on various developmental days.

Call types	Day 1		Day 5		Day 9		Day 11		Day 15		Day 21	
	Control	REMSR	Control	REMSR	Control	REMSR	Control	REMSR	Control	REMSR	Control	REMSR
**A**	34 (13, 58)	28 (11, 49)	33 (14, 46)	47 (19, 65)*	60 (22, 130)	43 (27, 84)	60 (13, 130)	63 (26, 100)	42 (29, 121)	41 (14, 52)	19 (14, 25)	38 (37, 54)
**B i**	14 (11, 17)	NF	15 (10, 34)	39 (12, 52)	22 (18, 69)	17 (13, 29)	26 (18, 36)	19 (14, 27)	18 (13, 29)	29 (20, 32)	14 (11, 23)	39 (26, 51)
**B ii**	34 (34, 34)	45 (32, 59)	35 (30, 69)	46 (33, 69)	75 (28, 121)	47 (25, 57)	71 (34, 137)	59 (36, 104)*	13 (11, 64)	35 (16, 53)	NF	60 (32, 88)
**B iii**	NF	NF	19 (19, 19)	29 (28, 31)	48 (43, 49)	NF	36 (15, 95)	18 (18, 18)	36 (36, 36)	NF	NF	NF
**B iv**	27 (27, 27)	NF	NF	28 (28, 28)	24 (10, 35)	26 (11, 38)	22 (13, 47)	15 (14, 25)	16 (10, 45)	30 (11, 60)	21 (14, 28)	25 (25, 25)
**C**	60 (50, 97)	NF	40 (33, 46)	71 (55, 90)	72 (13, 110)	46 (33, 93)	72 (13, 110)	63 (23, 89)*	38 (12, 89)	60 (32, 99)	21 (21, 21)	40 (22, 60)
**D i**	58 (58, 58)	NF	68 (68, 68)	68 (68, 68)	50 (40, 60)	63 (61, 66)	64 (38, 99)	51 (43, 93)	48 (19, 137)	47 (39, 54)	NF	62 (62, 62)
**D ii**	NF	NF	NF	NF	93 (64, 220)	311 (311, 311)	54 (51, 56)	NF	39 (39, 39)	74 (74, 74)	NF	NF

The values are presented in msec and are tabulated as median (minimum, maximum) in bracket. NF stands for calls that were ‘not found’. A denotes category consisting of flat calls; B having upwards (i), downwards (ii), U shaped (iii), and inverted-U shaped (iv); C is sum of 2 and 3 syllable and D contains wave (i) and complex calls (ii). The significant changes on comparison of calls between control and REMSR are depicted in ‘*’. The level of significance is *p*<0.05.

**Figure 6:**
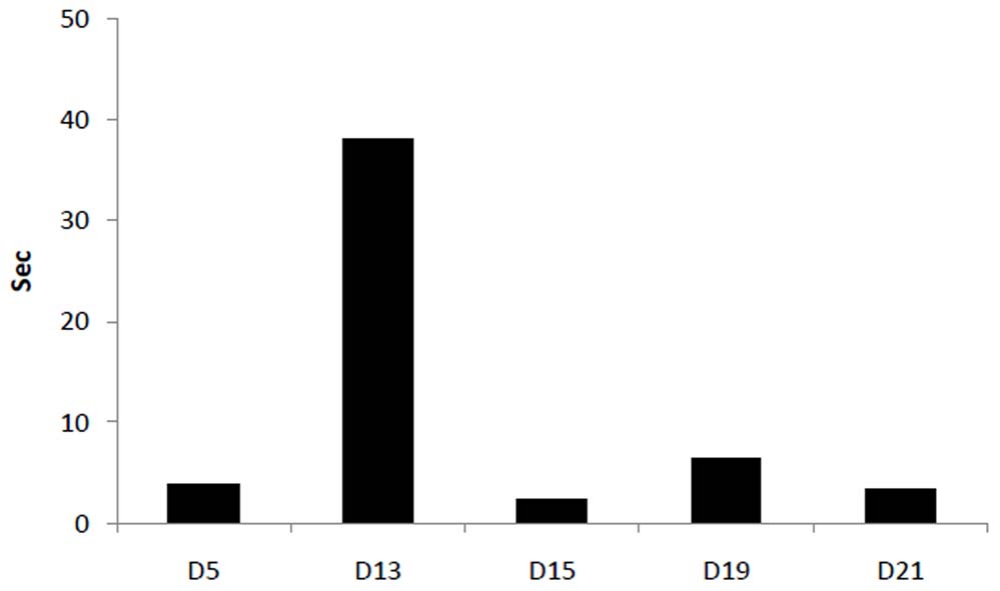
Plot of excess time spent in ultrasonic vocalization by the pups in sleep challenged mothers. Plot of excess time spent in ultrasonic vocalization by the pups in sleep challenged mothers (difference taken from total time spent in vocalization by control pups) during testing days in the isolation paradigm.

To compare total time spent in vocalization on various days, the difference in total time was calculated between the two groups. The excess time spent in calling, on the post-natal days 5, 13, 15, 19 and 21, by the pups of REMSR mothers is plotted in Figure 6.

### Fundamental frequency

The carrier/fundamental frequency in the flat calls in the control group pups remained around 40 kHz in majority of cases on day 5 (80%), days 9–11 (80–90%) and increased to higher frequencies on later days. Flat calls of higher frequencies were also observed on day 5 (45–46%) in the pups of REMSR mothers. The remaining MF calls were centred at higher frequency of 55–65 kHz in both the groups. The frequency overtones/harmonics in ultrasonic vocalizations were also common in both the groups.

### Amplitude

**Figure 7:**
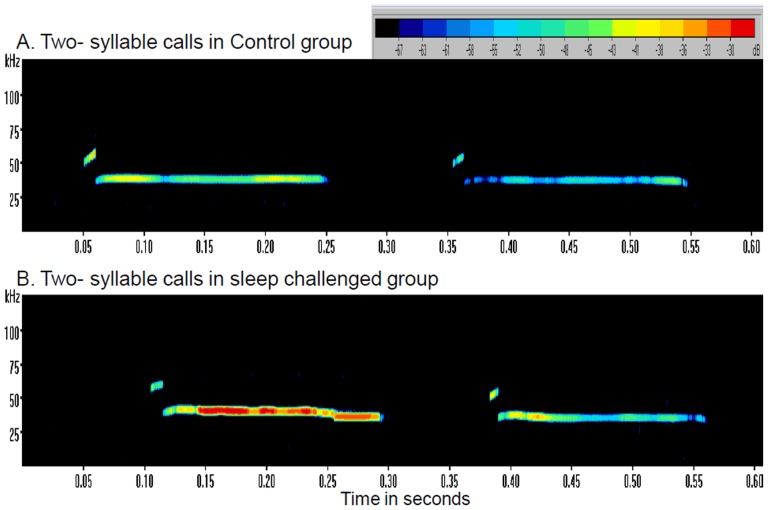
Representative intensities in 2-syllable ultrasonic calls made by pups in control and sleep challenged mothers. Sonograph of 2-syllable ultrasonic calls showing alteration in intensities (depicted in colour coding of increasing intensity from blue to red) obtained in control and the REMSR group pups on day 11.

The majority of calls made by pups in the REMSR group were lower in amplitude than the pups of the control group during the initial week. The CF calls (Flat) on day 13 were also significantly less intense than those of their control counterparts (*p*<0.02). Only MF 2 syllable calls in these pups on day 11 (*p*<0.04) and 15 (*p*<0.006) were higher in amplitude than in the control group (Figure 7). On day 7, majority of calls made by the pups of REMSR mothers were higher in amplitude (−34 to −57) than the control pups (−46 to −70).

### USV distribution over first and second min

The numbers of calls made during the initial first minute was higher till day 15 in control groups of pups. Similar trends were observed in pups from REMSR group except during initial (days 1–3) and later days (19–21) when a higher number of calls were observed during the second minute.

### Retrieval test

**Figure 8:**
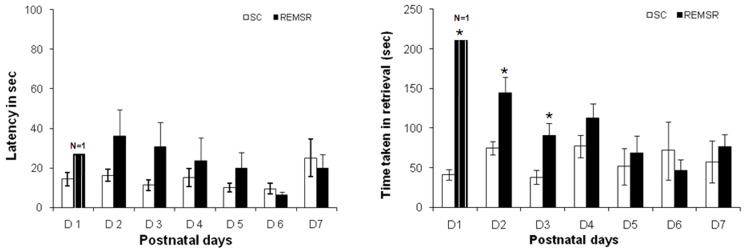
Latency of retrieval of first pup and time taken for retrieving all the pups on different postnatal days in control and REMSR group rats. Latency to retrieval of first pup is shown in A. The post-natal days are shown on horizontal axis and time in sec is shown on the vertical axis. The open bars depict Control and closed bars depict REMSR group data. The hatched closed bar depict retrieval by few mothers (such number of cases are written above the bar).

In comparison to control group mothers which showed normal retrieval behaviour, only one animal of REMSR group showed retrieval behaviour on day one. In addition, time taken in retrieving all the pups of the REMSR group was significantly higher in comparison to the pups of the control group on initial three days (Figure 8). Thereafter, a gradual increase in the retrieval response was observed. There was an increase in latency to retrieve the first pup in the REMSR group, though the trend did not reach the level required for statistical significance.

### Temperature

The body temperature of pups was 36.5±0.2°C when they were confined to mother's crouch on all the recorded days. When pups were huddled in nest with other pups, the temperature ranged between 33.4±0.3 to 34.2±0.2°C whereas when pups were lying scattered in the cage, temperature dropped to 30.1±1.2°C on initial 2 days, irrespective of the group that they belonged to.

## Discussion

The temporal profile of isolation induced distress calls produced by the control group pups on different postnatal days is comparable to earlier reports on some of the postnatal days [17], [37]. However, the present detailed study on different postnatal days showed that the USVs followed a distinct developmental pattern. In addition, the present study showed a profound change in USVs of pups born to the REM sleep-compromised mothers. These pups showed less number of USVs during initial days, with a significantly reduced calling rate during the second week of peak vocalization in comparison to controls. Their increased vocalization was shifted to the end of the 2^nd^ week. Strikingly lowered USVs in these pups of REMSR mothers during the second week (days 9 and 11), can be due to their reduced sensitivity to isolation stress or to a reduction in their ability to call. A poor display of retrieval response by these REMSR mothers during the initial crucial days (days 1–3) and a consequent drop in body temperatures of pups when lying scattered could have contributed to the change in their vocalization response on the subsequent days. The diminished response in experimental pups on isolation strongly indicates a potential link between expression of USVs and maternal sleep.

It is claimed that the distress induced vocalizations emitted by neonates are the acoustic signals for their mother to carry out an appropriate retrieval response [38]–[40]. Reduced vocalization in pups would result in reduced care giving response by mothers. A weaker calling response during initial post-natal days can be attributed to comparatively reduced sensitivity to hypothermia. During the subsequent days, pup's vocalizations increase, inducing retrieval behaviour in their mothers, coinciding with the period of development of thermoregulation [15]. Finally, during the end of 3^rd^ week, ultrasonic vocalizations nearly cease with the growth of fur in pups. However, the extreme reduction in calling rates of REMSR group pups on peak vocalization days suggests that they had different levels of sensitivity and responsiveness to the maternal isolation which could be contributed by prenatal stress as a result of maternal sleep restriction during pregnancy. This is supported by another report that demonstrated that prenatal stress is indicated to suppress USVs in pups [41]. This might probably also reflect an early onset of a distorted/malformed social bonding with their mothers.

The second peculiar observation in this study was an altered vocalization pattern in the pups born to REM sleep-compromised mothers. Though these pups produced a lower number of ultrasonic vocalizations on peak calling days (days 9–11), unlike the control pups, their calling rate did not show a decrease on day 13. This extended vocalization response, and inability of these pups to reduce ultrasonic calls prior to weaning (day 21) possibly reflects a subtle developmental delay produced by REMSR. These results strongly suggest an early onset of anxiety behaviour in these pups which is supported by evidences from previously published work [30], [42]. Report of reduced USVs on postnatal day 9 in the vasopressin 1b knockout mice shows the vulnerability of ultrasonic vocalizations, and possibly social bonding, during early development [43].

An evaluation of qualitative aspects of the USVs provided further details on their distribution across development. Since the isolated dot calls did not differ between the two groups on different developmental days, it can be assumed that these might not have a specific role in the current paradigm. Predominance of flat calls during the initial days of post-natal development indicates that pups are capable of making only simpler constant frequency calls during initial days. The ability to produce frequency modulated calls gradually increased during the later days of growth. However, it can be seen that the pups from REMSR group made more efforts in making larger number of calls in both constant and high pitched single/multiple syllable frequency modulated calls on day 5 (Figure 4). In addition, these pups of REM sleep-compromised mothers made longer duration calls on day 5 which however was lacking during the further days of development. This indicated that initially these pups attempted to vocalize for a longer time supplemented with high pitched sounds. But they discontinued their effort in absence of an optimal care response from their mother. This may be responsible for their lower calling rate on day 5. The trend for an increased count in the 2–3 syllable calls which display higher frequency modulation is suggestive of increased signs of anxiety in pups born to the REM sleep-deprived mothers. The reduced numbers of complex and wave types of calls in pups whose mothers were REM sleep- deprived also indicate a reduction in their ability to produce these frequency modulated calls. The increased time in vocalization on day 13 onwards, in pups of REM sleep deprived- mothers, is due to the persistent, but delayed, increase in calling rate. The control group pups generally showed reduction in calling rate from day 11 onwards.

Increase in duration and calling rate of flat calls, and existence of the higher frequency calls in REMSR pups on day 5, probably suggests that these pups tried high pitch modality during early development. The reduction in rate of downward calls in these pups indicates that the flat calls may be easier to manoeuvre. The lower number of flat, downward, inverted-U and 2 syllable calls in the pups of REM sleep-compromised mothers on day 11 indicate reduction in both the constant and frequency modulated calls.

Increased total time of low intensity USVs on day 5 in pups of REMSR mothers is probably suggestive of an attempt to attain mother's attention in spite of making less number of calls. The reduction in calling rates, and vocalization time in these pups on day 9–11 might be suggestive of their reduced sensitivity to isolation stress. Recent study provides evidence that the specific patterns of USVs may be mirroring alteration in emotional state in rat pups [36]. The factors like malnutrition during the prenatal period can also contribute to abnormal USVs in pups without altering the calling rates and variety of calls [37].

An altered crying pattern with abnormal cries is related to brain damage in human babies [12]. It is thus possible that pup ultrasonic vocalizations that are altered in the absence of an optimal environment during early postnatal period, might manifest in emotional deficits during later life. The exact message hidden in the individual call types are not yet deciphered but it would be interesting to decode these acoustic signals to understand importance of the vocalizations. These conclusions are supported by the reports that the USVs of the pups shape the mother's responsiveness towards the pups [40]. In contrast, the USVs emitted by adults display more distinct acoustic features than the pups and are largely grouped into two broad categories. The long 22-kHz USVs are emitted in aversive conditions such as on exposure to painful stimulus, after defeat in aggressive encounter, after ejaculation during mating whereas the short 50-kHz calls are emitted during copulatory act, play with mates and positive motivational states [22], [26]–[28].

The present findings reiterate that isolation calls in pups vary in carrier frequency and display change in the sonographic structure during development. Quantitative and qualitative alterations in USVs of pups may be modulating the maternal retrieval response. The mechanism and expression of anxiety might vary across various phases of ontogeny (neonatal, adolescent, adult and old age) which could be defined by specific experiences in age dependent manner for emotional learning. However, during early development, distress giving variables may be limited and common to all pups. Since evaluation and assessment of newborn's behaviour is comparatively difficult, it is emphasized that the USVs measurement in neonates can be taken as a possible tool to study various indices of emotional development [28], [37].

The disturbance in REM sleep is associated with depression in mothers [2]. The results of the current study support recent human studies associating maternal stress with adverse neural developmental in children [44]. It is difficult to assert from the present study that the alterations in USVs were due to prenatal factors alone and it did not have a contribution from postnatal factors. Though the brain development during postnatal period is more important in determining cognitive function [45], the impaired neural development during foetal life may increase the susceptibility to emotional development [44], [46].

## Conclusion

The results of the present study suggest the importance of maternal REM sleep in modulation of USVs of the neonates born to them. The pups born to REM sleep-compromised mothers not only showed qualitative changes in calling rates but also displayed an altered vocalization pattern during development. Reduction in vocalization response to isolation on peak calling days, variations in the types and characteristics of call types, and alteration in temporal profile, together highlight the importance of maternal sleep in shaping the emotional behaviour in neonates.

It is reasonable to assume that the ultrasonic vocalizations can be utilized as a reliable early marker for an affective state in the rat pups and this model could be used to understand the mother-child bonding for an optimal cognitive development during post-partum life. These findings may have an important clinical implication in human neonates, as delayed vocalization may be associated with REM sleep deprivation in mothers during late pregnancy. It would be interesting to see whether reduced USVs on the peak calling days has any relationship with depression traits. Further studies are required to decode USVs to understand plasticity in the pups' vocalization and the maternal responses in shaping their behaviour during post-natal period. This is the first report showing a link between maternal REM sleep deprivation and the vocalization in neonates and infants.
